# Real-time contrast-enhanced ultrasound-guided percutaneous biopsy in the diagnosis of ovarian metastasis of gallbladder carcinoma: a case report

**DOI:** 10.1186/s13048-023-01198-y

**Published:** 2023-07-07

**Authors:** Jing Wang, Yanjun Liu, Liang Sang, Weina Wan

**Affiliations:** Department of Ultrasound, The First Hospital of China Medical University, People’s Republic of China, Shenyang, 110001 Liaoning Province China

**Keywords:** Contrast-enhanced ultrasound (CEUS)-guided, Percutaneous biopsy, Gallbladder carcinoma, Krukenberg tumor

## Abstract

**Background:**

Multiple-organ primary tumors can invade the ovary through lymphatic and hematogenous routes, presenting as ovarian Krukenberg tumors, but these rarely originate from the gallbladder. Krukenberg tumors can present similar to primary ovarian tumors; however, their treatments are completely different.

**Patient concerns:**

A 62-year-old Chinese woman presented with abdominal distension for six months and weight loss of five kilograms for two months.

**Diagnoses:**

Based on multiple imaging examinations, the patient was preliminarily diagnosed with a malignant tumor of unknown origin with multiple metastases (omentum). To identify the origin of the malignancy, the patient underwent real-time contrast-enhanced ultrasound-guided percutaneous biopsy. The results revealed a perihepatic hypoechoic lesion and right adnexal mass that were both metastatic adenocarcinomas from the gallbladder.

**Interventions:**

The patient initially received chemotherapy with gemcitabine and cisplatin instead of surgery. However, the tumor increased in size on re-examination after two cycles, so the treatment was shifted to a combination regimen with durvalumab for six cycles.

**Outcomes:**

The treatment proceeded smoothly, with no recurrence or obvious progression of the cancer during follow-up.

**Conclusions:**

Differentiating between primary and metastatic ovarian tumors is important. Early diagnosis and effective treatment options are essential for patient survival. CEUS-guided percutaneous biopsy is a valuable procedure for patients with multiple metastases who cannot tolerate surgery.

## Background

A Krukenberg tumor is a rare metastatic ovarian malignancy, and most tumors of this kind originate from the gastrointestinal tract, such as the stomach and colon. However, cases originating from the gallbladder or hepatic biliary duct are less widely reported [1]. This type of tumor usually occurs bilaterally, and only a few unilateral cases have been observed [2]. We reported a rare case of metastatic right ovarian malignancy originating from the gallbladder and discussed our analysis of the relevant imaging findings, diagnostic process, and treatment methods.

## Case presentation

A 62-year-old Chinese woman presented with abdominal distension for six months and weight loss of 5 kg for two months. The patient had a history of hypertension, coronary disease, and diabetes and denied any abnormal vaginal discharge or bleeding. Her physical examination revealed an immobile egg-sized mass, exerting painful pressure, located on the right side and posterior to the uterus. Laboratory tests revealed a carbohydrate antigen (CA) 125 level of 135.00 U/mL, CA 199 level of 651.00 U/mL, and CA 724 level of 10.30 U/mL, all of which were elevated compared to normal levels.

**Fig. 1:**
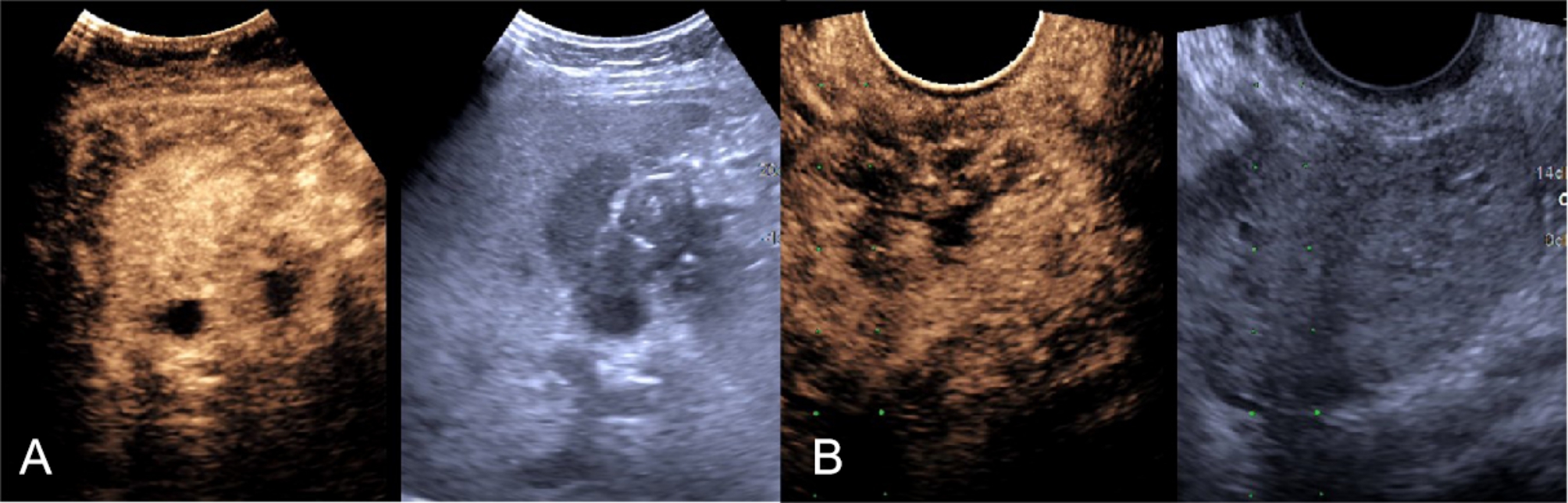
Ultrasound examination. (**A**) contrast-enhanced ultrasound (CEUS) showed that the gallbladder and intrahepatic hypoechoic lesion were enhanced earlier than the other parts of the liver. (**B**) an irregularly substantial mass was seen in the right area which showed uneven high enhancement with rapid advancement and rapid exit on CEUS

Gynecologic ultrasonography (US) (Fig. 1) revealed an irregular mass, 4.4 × 3.8 cm in size, with rich blood flow in the right adnexal area. Abdominal US showed a thickened and partially indistinct gallbladder wall and an uneven hypoechoic lesion, 3.3 × 2.5 cm in size, in the gallbladder cavity. The liver tissue adjacent the gallbladder displayed irregular hypoechogenicity.

**Fig. 2:**
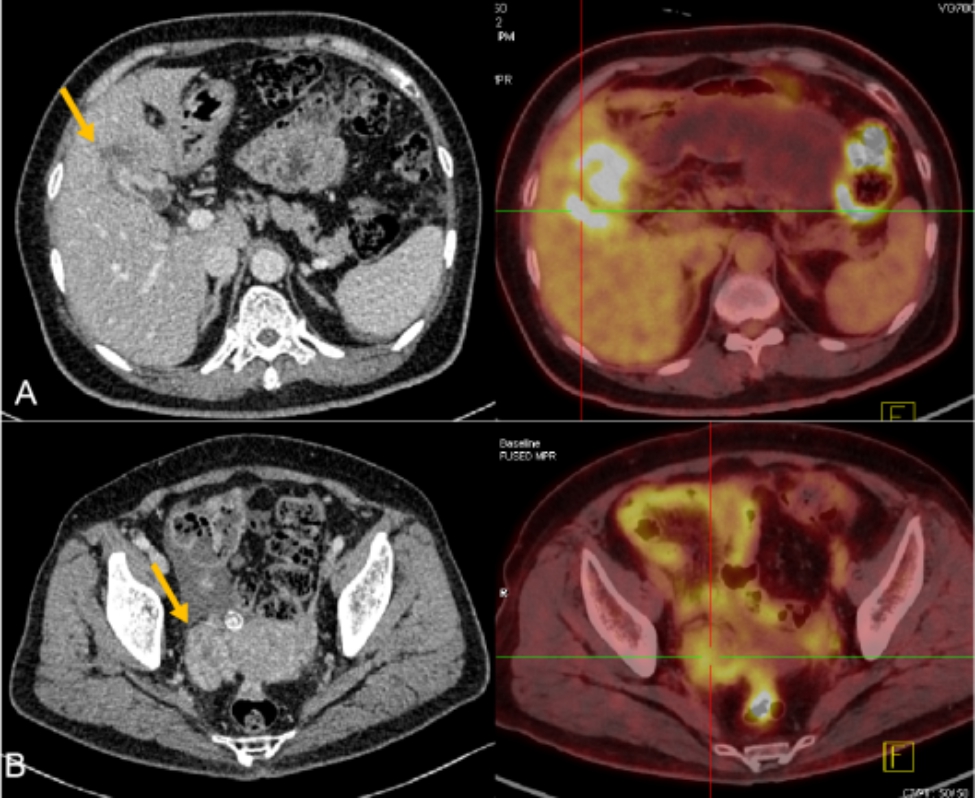
Computed tomography (CT) and positron emission tomography (PET)-CT imaging. (**A**) PET-CT showed increased fluorodeoxyglucose (FDG) uptake in the fossa of the gallbladder and adjacent liver tissue, with a maximum standardized uptake value (SUV) of 8.2. CT showed enhancement of the thickened gallbladder wall and a hypodense lesion in the liver with ill-defined boundaries. (**B**) PET showed increased FDG uptake in the right adnexal area with a maximum SUV of 5.8, and CT showed soft tissue nodules

Enhanced abdominal computed tomography (CT) revealed a thickened and irregular gallbladder wall with internal malignant changes and involvement of the adjacent liver tissue. The right adnexal area showed uneven enhancement of a mass, which was suspicious for malignancy. In addition, CT revealed fluid densities in the abdominal and pelvic cavities. Positron emission tomography (PET)-CT was performed for further evaluation and revealed metabolic enhancement of not only the right ovarian mass, gallbladder, and hepatic tissue, but also the omentum, intestinal canal, abdomen, and pelvic cavity (Fig. 2).

**Fig. 3:**
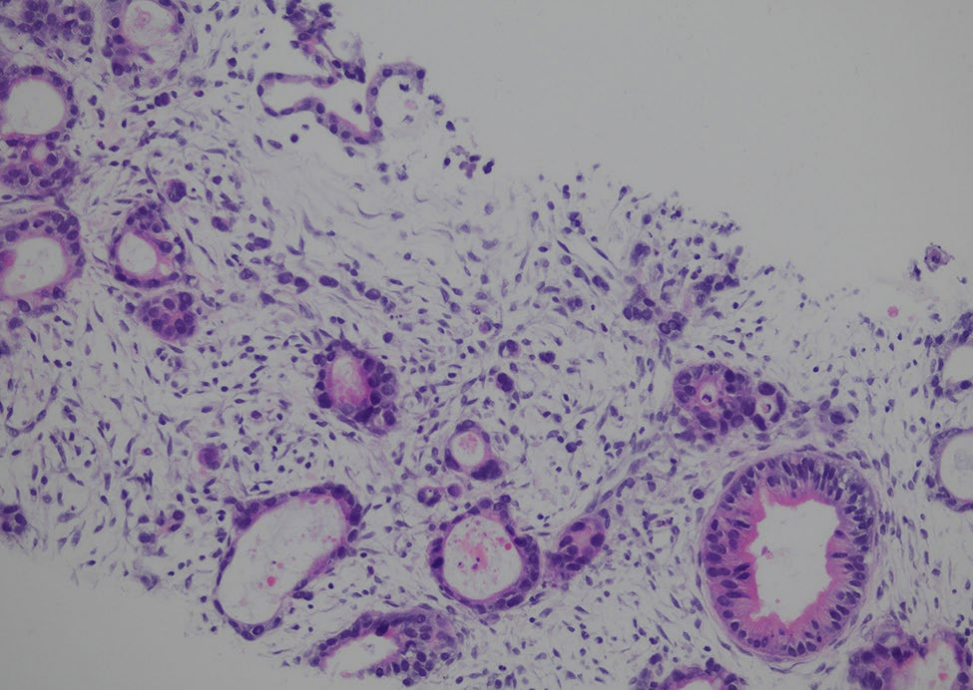
Histopathologic image (x200). Tumor cells were arranged in a glandular tubular pattern with signs of invasive growth

**Fig. 4:**
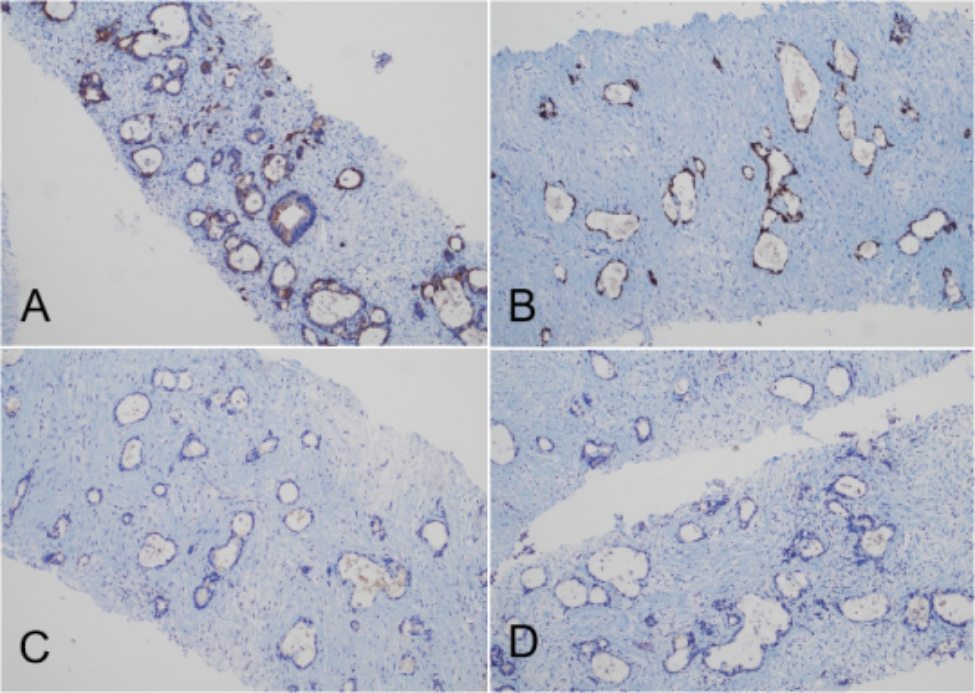
Immunohistochemical staining (x100). (**A**) Cytokeratin 7(+); (**B**) Caudal type homeobox 2 (+); (**C**) Pair box gene 8(-); (**D**) Special AT-rich binding protein 2 (-)

Based on these examination results, the patient was preliminarily diagnosed with a malignant tumor of unknown origin with multiple metastases and received multidisciplinary treatment. To identify the origin of the malignancy, the patient underwent a real-time contrast-enhanced ultrasound-guided (CEUS-guided) percutaneous biopsy, which revealed a perihepatic hypoechoic lesion and right-adnexal mass that were both metastatic adenocarcinomas from the gallbladder (Fig. 3). Immunohistochemical tests showed positive expression of caudal type homeobox 2 (CDX2), cytokeratin 7 (CK7), cytokeratin 20 (CK20), and Ki-67 (60%+) markers and negative expression of special AT-rich binding protein (SATB2) and pair box gene 8 (PAX8) markers (Fig. 4) [3].

Because our patient had multiple metastatic lesions accompanied by comorbidities such as hypertension, coronary disease, and diabetes, she underwent chemotherapy with gemcitabine and cisplatin instead of surgery. However, the outcome was poor, and she was regularly followed up.On July 5,2022,our patient received chemotherapy with GP regimen(Three weeks of therapy, gemcitabine 1.8 g D1 D8,platinum 42 mg D1 D8).On July 13,2022,the dose was adjusted for patient’s weight change(Three weeks of therapy, gemcitabine 1.6 g D1 D8,platinum 40 mg DI D8).After two courses,the lesion increased in size on re-examination, and the tumor marker value increased.therefore, the treatment was shifted to a combination regimen with durvalumab from August 18,2022 for 6 courses(gemcitabine 1.6 g D1/D8,platinum 40 mg D1/D8,dovaliuzumab 1000 mg D1).The treatment proceeded smoothly, with no recurrence or obvious progression of the cancer during follow-up.

## Discussion

Metastatic ovarian cancer often has its origins from various organs and represents approximately 5–15% of all ovarian tumors [4–6], Krukenberg tumors account for only 1–2% of these metastatic tumors [7] and are often confused with primary ovarian tumors and teratomas. Most patients with gallbladder-derived ovarian malignant tumors have no obvious and specific clinical manifestations, often manifesting with abdominal pain or distension, and only a few patients develop biliary symptoms, such as jaundice [8, 9]. In the patient in our case, only abdominal distension was present as the first symptom, with a pelvic mass palpated during physical examination. The patient also had insidious abdominal pain that was initially considered to be gastritis. Ascites, which usually presents in advanced stages of cancer, was also observed.

No clear differences between primary and metastatic ovarian tumors have been identified from current imaging reports [2]. However, imaging examinations are still valuable in the diagnosis of ovarian metastatic tumors. First, imaging examinations can detect lesions outside the accessory area, thus increasing the probability of the diagnosis of metastatic ovarian cancer. Preoperative US, CT, and PET-CT can also evaluate disease progression and provide guidance for treatment. Second, it has been reported that metastatic ovarian cancer is usually bilateral and more common on the right side [2]. Therefore, in the presence of bilateral tumors, other organs are often examined to determine the presence of a primary malignancy. Metastatic ovarian tumors often present as solid or mixed cystic masses, whereas primary ovarian tumors often present as cystic masses or masses with liquefied necrotic areas [10]. The imaging results in our case revealed a solid primary mass, which is different from the presentation of primary ovarian cancer. However, it appeared as a unilateral ovarian mass, which increased the difficulty of diagnosis. These imaging results were useful for establishing systemic metastasis but lacked specificity. Thus, we performed a real-time CEUS-guided percutaneous biopsy to identify the primary lesion. Percutaneous biopsy is considered conclusive in the diagnosis of cases such as this.

Enhanced ultrasound can clearly delineate tumor blood vessels and necrotic lesions; therefore, we selected contrast-enhanced sites to optimize the positivity rate for percutaneous needle biopsy [11, 12]. CEUS-guided percutaneous biopsy also allowed us to monitor the position of the biopsy needle to avoid damaging the tissue and blood vessels around the mass [13]. After injection of contrast media, the lesion began to strengthen earlier than the uterine muscle wall which showed uneven high enhancement. Large blood vessels were seen in the lesion entering the interior from the side of the mass. A weakly enhancing area inside the mass was observed and considered necrotic or liquefied; therefore, during the puncture process, we avoided the central necrotic area and selected the enhancement area to obtain positive results.

Metastatic ovarian tumors are very difficult to distinguish from primary tumors; however, it is important to distinguish between them because their corresponding treatments are quite different. Differential diagnosis often relies on the patient medical history and immunohistochemical examination. CK7 and CK20 are important markers for distinguishing ovarian tumors [14]. CDX-2 is highly expressed in gallbladder cancer, while it is negative in primary ovarian cancer. SATB2 is highly expressed in the lower epithelial tissues of the digestive tract, and the negative expression of PAX8 can clearly exclude a primary tumor and support a metastatic origin [15].

At present, there is no standard or consensus on the treatment method for metastatic ovarian cancer. Appropriate surgical intervention may prolong survival in patients with primary tumors, but it is not as effective in patients with metastatic tumors that require combination regimens involving radiotherapy or chemotherapy. Moreover, primary ovarian tumors are typically sensitive to platinum, while gallbladder cancer often requires a combination regimen that includes gemcitabine [16]. However, because of the poor prognosis of the patient in our case and the increase in mass size and tumor marker levels despite two courses of gemcitabine and platinum drugs, we switched to combination therapy with durvalumab for six courses, and the treatment results were shown to be effective.

## Conclusion

The case we report is a rare presentation. First, metastatic tumors of the ovary, especially those originating from the gallbladder, are extremely rare. Second, metastatic ovarian cancer is typically bilateral, whereas the patient in our case had unilateral metastasis, which increased the difficulty of diagnosis. CEUS-guided percutaneous biopsy is a valuable procedure for patients with multiple metastases who cannot tolerate surgery. Although medical history and imaging examination can help rule out primary ovarian cancer, the definitive diagnosis is still based on histopathological examination.

### Manufacturer information

Gemcitabine (Zhejiang Hisun Pharmaceutical); Platinum (QiLu Pharmaceutical); Dovaliuzumab (AstraZeneca UK).

## Data Availability

The data presented in this study are available on request from the corresponding author. The data are not publicly available due to restrictions of patient privacy.

## Declarations

### Data access statement

The research data supporting this publication is available from the NN repository at www.NNN.org/download/.

## Abbreviations

CEUS: contrast-enhanced ultrasound-guided
CA: carbohydrate antigen
US: ultrasonography
CT: computed tomography
PET-CT: positron emission tomography-computed tomography
CDX2: caudal type homeobox 2
CK7: cytokeratin 7
CK20: cytokeratin 20
SATB2: special AT-rich binding protein
PAX8: pair box gene 8
FDG: fluorodeoxyglucose
SUV: standardized uptake value
